# Using medical comics to highlight medical humanities

**DOI:** 10.1111/medu.14308

**Published:** 2020-09-22

**Authors:** Eva Katharina Masel, Anna Kitta, Ruth Koblizek, Andrea Praschinger

## 1 WHAT PROBLEMS WERE ADDRESSED?

Studying medicine comprises the extensive acquisition of knowledge, skills and attitudes. However, humanism in medicine, which combines scientific knowledge and skills with respectful, compassionate care that is sensitive to the values, autonomy, and cultural backgrounds of patients and their families, is often neglected by teachers, physicians, medical students and the medical curricula. However, a humanistic approach is indispensable in the medical field.

## 2 WHAT WAS TRIED?

By creating a novel and remarkable initiative for the medical humanities, an attempt was made to increase the attention that is paid to this under‐addressed topic and to stimulate debate. As an interdisciplinary multifaceted field, the medical humanities apply numerous approaches, one of which is medical comics. Graphic illustrations have the potential to address challenging situations within medical settings.^1^ They can be used to illustrate the different perspectives of patients, caregivers, relatives and the medical staff. Insightful comics prompt people to pause and reflect.

An exhibition of 22 medical comics, named ‘Impression – Expression – Interaction', was displayed freely accessible at the Medical University of Vienna’s Lecture Center in Vienna General Hospital from October 2019 until January 2020. The artwork was supplemented by additional information, reflection‐based tasks and recent publications.

## 3 WHAT LESSONS WERE LEARNED?

After overcoming initial reluctance towards the word ‘comics’, primarily associated with ‘fun’ and ‘humour’, support was offered by various executives (eg the Vice Rector for Teaching at the Medical University and the Director of the Vienna General Hospital). The artists were extremely cooperative and agreed to have their work exhibited. A professional graphic designer was commissioned to produce premium‐quality panels and additional material (eg invitation posters and booklets), particularly as the first impression of a comic is very important as it stimulates introspection. In the exhibition area, visitors could provide prompt feedback via a tablet. Seventy‐eight per cent of the respondents reported that the exhibition had introduced them to a new topic with which they were previously unfamiliar. More importantly, 84% indicated that they had acquired a new perspective on a familiar topic. On average, the exhibition was rated 5.34 (range 0–6, with 6 as the highest rating). Participants in the guided tour reported that the comics made it easier for them to approach challenging and painful topics, such as grief, death and excessive demands during night shifts. Positioned alongside the comics, an interactive zone invited visitors to provide their own drawings on different topics using prompts such as ‘What does pain look like?’. Their creations (165 contributions) were then displayed for viewing.

The immensely positive feedback to this unique approach inspired the establishment to an ongoing three‐year project—including an exhibition of medical comics every winter term starting in fall 2020 at the premises of Medical University in Vienna’s Lecture Center in the General Hospital. In addition, medical comics will now be integrated step‐by‐step in compulsory teaching units in different fields of study at the Medical University of Vienna. Cooperation with other universities and teaching institutions is planned.
